# Alterations of Gastric Microbiota in Gastric Cancer and Precancerous Stages

**DOI:** 10.3389/fcimb.2021.559148

**Published:** 2021-03-03

**Authors:** Xinmei Zhang, Chao Li, Weijun Cao, Zhenyu Zhang

**Affiliations:** ^1^Department of Gastroenterology, Nanjing First Hospital, Nanjing Medical University, Nanjing, China; ^2^Department of Gastroenterology, Jiahui International Hospital, Shanghai, China

**Keywords:** gastric carcinogenesis, microbiota, taxonomic biomarker, gastric cancer, gastric cardia cancer, gastric intraepithelial neoplasia

## Abstract

**Objective:**

Microbial infections have been shown to contribute to gastric carcinogenesis, the knowledge of gastric microbiota alteration in this process may provide help in early diagnosis of gastric cancer. The aim of this study was to characterize the microbial changes and identify taxonomic biomarkers across stages of gastric carcinogenesis.

**Methods:**

The gastric microbiota was investigated by 16S rRNA gene analysis in gastric mucosal specimens from 47 patients including superficial gastritis (SG), atrophic gastritis (AG), gastric intraepithelial neoplasia (GIN), and gastric cancer (GC). Differences in microbial composition across the disease stages, especially in GIN and GC were assessed using linear discriminant analysis effect size.

**Results:**

There was no gradual changing trend in the richness or diversity of the gastric microbiota across stages of gastric carcinogenesis. The relative abundance of dominant taxa at phylum and genus levels didn’t show a gradual shift pattern, and the only four taxa that continuously enriched from SG to GC were *Slackia*, *Selenomonas*, *Bergeyella*, and *Capnocytophaga*, all of which were oral bacteria. The most representative taxa which were enriched in GC patients were oral bacteria including *Parvimonas*, *Eikenella* and *Prevotella-2*, and environmental bacteria including *Kroppenstedtia*, *Lentibacillus*, and *Oceanobacillus*. The gastric microbiota in GIN patients were characterized by enrichment of intestinal commensals including *Romboutsia*, *Fusicatenibacter*, *Prevotellaceae-Ga6A1-group*, and *Intestinimonas*. Gastric cardia cancer and non-cardia cancer patients had significantly different microbiota profiles characterized by a higher abundance of *Helicobacter* in the cardia cancer patients.

**Conclusions:**

Our results provide insights on potential taxonomic biomarkers for gastric cancer and precancerous stages, and suggest that gastric microbiota might play different roles in the carcinogenesis of cardia cancer and non-cardia cancer.

## Introduction

Gastric cancer remains the second-leading cause of cancer-related deaths worldwide, which emphasizes the need for knowledge of the key changes in the process of carcinogenesis to make early diagnosis. According to Lauren’s histological classification, gastric cancer is classified into the diffuse type and the intestinal-type (Lauren, 1965). The current widely accepted biological model of intestinal-type gastric carcinogenesis can be described as a series of sequential stages: from superficial gastritis (SG) through atrophic gastritis (AG), intestinal metaplasia (IM), and gastric intraepithelial neoplasia (GIN) to gastric cancer (GC) (Correa, 2004). Other than genetic predisposition, environmental factors including microbial infections have been shown to contribute to gastric carcinogenesis. Chronic *Helicobacter pylori* (*H. pylori*) infection is considered to be the strongest risk factor for intestinal-type gastric cancer, but only 3% of people with *H. pylori* infection develop gastric cancer, implying that other factors also play a role (Björkholm et al., 2003).Studies have presented evidence that gastric carcinogenesis can be associated with enrichment in many non-*H. pylori* bacteria, as well as depletion in others (Emanuel et al., 2016), although there are inconsistent conclusions about the changing trend of a specific bacterium. The changes of gastric microbiome in the development of gastric cancer remains largely unknown. Previous studies have revealed a gradual shift in gastric microbiota profile from SG to IM to GC (Coker et al., 2018; Eun et al., 2014; Francisco et al., 2014). Gastric intraepithelial neoplasia, including low-grade and high-grade intraepithelial neoplasia, is the precancerous lesion of gastric cancer, and mainly develops based on atrophic gastritis with intestinal metaplasia. The prognosis of patients with gastric cancer can be greatly improved by early diagnosis and endoscopic resection of GIN. So far, the microbial profile of GIN in the process of gastric carcinogenesis is barely known. In this study, we investigate the microbial changes in mucosal biopsies of patients with progressive histological stages along gastric carcinogenesis—from SG, through AG and GIN to GC.

## Materials and Methods

### Patients

Gastric biopsies were collected from 47 patients during upper gastroenterology endoscopic examination or endoscopic submucosal dissection (ESD) due to precancerous mucosal lesions from August 2016 to March 2019 at Nanjing First Hospital, China. The pathological diagnosis was confirmed for each patient, and the results showed 17 patients of superficial gastritis (SG group), 10 patients of atrophic gastritis with intestinal metaplasia (AG group), five patients of gastric intraepithelial neoplasia (GIN group, including four patients of low-grade GIN and one patient of high-grade GIN), and 15 patients of intestinal-type gastric cancer (GC group), including 27 male patients and 20 female patients in total. For pathological diagnosis, two biopsies were taken from antrum and corpus in SG and AG group, if both tissues showed superficial gastritis, then it was classified into SG group, if atrophic gastritis was seen in either site, then it was classified into AG group. GIN specimens were obtained through ESD, GC was confirmed by the specimens from the lesion. For microbiological analyses, two mucosal biopsies were obtained from antrum for SG and AG, and biopsies were taken from relatively intact mucosa adjacent to the lesion, other than necrotic tissue on the lesion, for GIN and GC. The biopsies were stored at −80°C. The age of the patients in each group was increasing from SG to CG (56.00 ± 10.25, 63.58 ± 6.69, 64.80 ± 9.93, and 69.87 ± 11.57 for SG, AG, GIN, and GC groups, respectively, *p*=0.005). This study was approved by the Ethics Committee of Nanjing First Hospital. Written informed consent was obtained from all of the patients in accordance with the Declaration of Helsinki.

### DNA Extraction

Bacterial genomic DNA was extracted with the E.Z.N.A ^®^Stool DNA Kit (Omega Bio-tek, Norcross, GA, U.S.), following the manual (**Supplementary Text**). The extracted DNA specimens were checked by NanoDrop for purity and quality, and stored at - 20°C.

### PCR Amplification

The variable regions 3 and 4 of the bacterial 16S rRNA gene were amplified from purified DNA specimens with the primers 338F (5’-ACTCCTACGGGAGGCAGCAG-3’) and 806R (5’-GGACTACNNGGG TATCTAAT-3’). PCR was carried out on a Mastercycler Gradient (Eppendorf, Germany) using a reaction mixture containing 12.5 μl 2× Taq PCR MasterMix, 3 μl BSA(2ng/μl), 1 μl Forward Primer (5 uM), 1 μl Reverse Primer (5 uM), 30 ng DNA with ddH_2_O to make up to 25 μl, 12.5 μl 2×Taq Plus Master Mix, 3 μl BSA (2ng/μl). PCR cycling parameters were a 5-min denaturation cycle at 95°C, followed by 32 cycles of the following: 95°C for 45 s, 55°C for 50 s, and 72°C for 45 s, and finally a 10-min extension at 72°C. Three PCR products per specimen were pooled to mitigate reaction-level PCR biases. The PCR products were purified using QIAquick Gel Extraction Kit (QIAGEN, Hilden, Germany) and checked using 1% agarose gel electrophoresis.

### Sequencing Processing

Paired-end sequencing was performed on Illumina Miseq PE300 platform (Illumina, San Diego, CA, USA), at Allwegene Company (Beijing, China). The raw data were filtered with QIIME (v 1.8.0), discarding the reads which were dereplicated or shorter than 150 bp. Filtered reads were clustered into operational taxonomic units (OTUs) assuming 97% similarity. Compared with the SILVA database (version 128), the species classification information of each OTU was obtained.

### Data Analysis

Rarefaction curves were calculated using Mothur (version 1.31.2) and R language to determine sequencing depth (Wang et al., 2012). Alpha diversity analysis (including the indexes of Chao 1 and Shannon, which reflect the richness and biodiversity of gastric microbiota, respectively) was performed using QIIME (Caporaso et al., 2010). The dissimilarity of the microbial communities among groups was evaluated by partial least squares discrimination analysis (PLS-DA) using R (Li et al., 2012). Sample clustering in beta diversity analysis was tested using analysis of similarity (ANOSIM) using the vegan package in R (Ferreira et al., 2018). The heatmap of genus information was also created using the vegan package in R. Linear discriminant analysis (LDA) effect size (LEfSe) was applied to find significant metagenome markers of different groups (Xu and Jiang, 2017). Only taxa with LDA >2 at a *P* value <0.05 were considered significantly enriched.

Statistical significance among groups was determined by Students’ t-test and one-way analysis of variance. Wilcoxon tests and Kruskal-Wallis rank tests were conducted in R to compare data between each two groups. A *P*-value less than 0.05 was considered to be significant.

## Results

### Quality Control and Basic Analysis

After sequencing and quality filtering, more than 2.3 million tags and a total of 3,055 OTUs were obtained (**Supplementary Table 1**). The dominant length of tags was among 400-440bp, and the average number of OTUs was 473 per specimen. The average number of reads per specimen in the SG, AG, GIN, and GC groups was 460,588,423 and 428, respectively.

### Richness and Diversity Analysis

**Figure 1:**
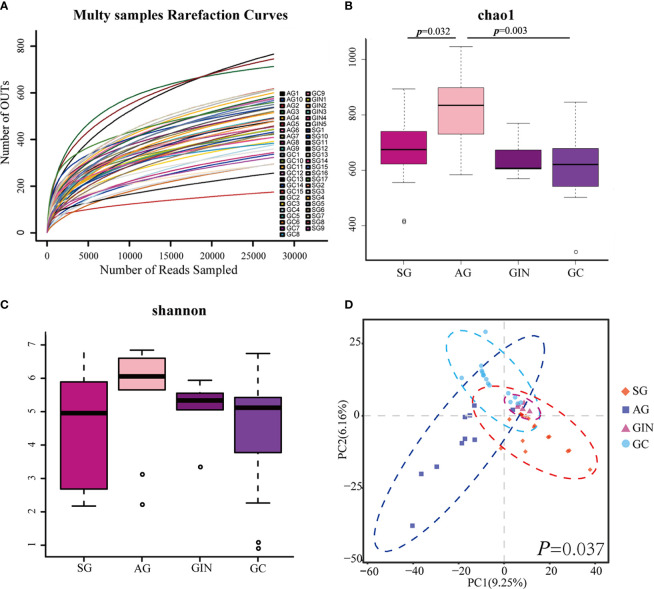
The microbial α diversity and β diversity analysis in different groups. **(A)** Rarefaction curves for operational taxonomic units (OTUs). **(B)** Chao 1 index was higher in atrophic gastritis (AG) compared with superficial gastritis (SG) and gastric cancer (GC) and **(C)** there was no significant difference in Shannon index among the four groups (Student’s t-test was used to compare each two groups). **(D)** Partial least squares Discriminant Analysis revealed different microbial community structures in the four groups (ANOSIM was used to evaluate the similarity among groups).

The microbial α diversity and β diversity were measured to analyze the differences of microbiota structure among the four groups. Firstly, to test the sequencing depth, we created the rarefaction curves and showed a reasonable amount of sampling (**Figure 1A**). The Chao1 index and Shannon index were used to describe the α diversity of the bacterial community results. We found that the richness of microbiota was significantly higher in AG group than in SG and GC group (Chao1 index, *p*=0.032 and 0.003, respectively, **Figure 1B**) and there was no significant difference in microbial diversity among groups (Shannon index, **Figure 1C**). Partial least squares Discriminant Analysis (PLS-DA) at the OTU level revealed a statistically significant separation of the groups (ANOSIM, *p* = 0.037; **Figure 1D**), suggesting different microbial community structures.

### The Changes of Gastric Microbiota Composition in Different Stages of Carcinogenesis

**Figure 2:**
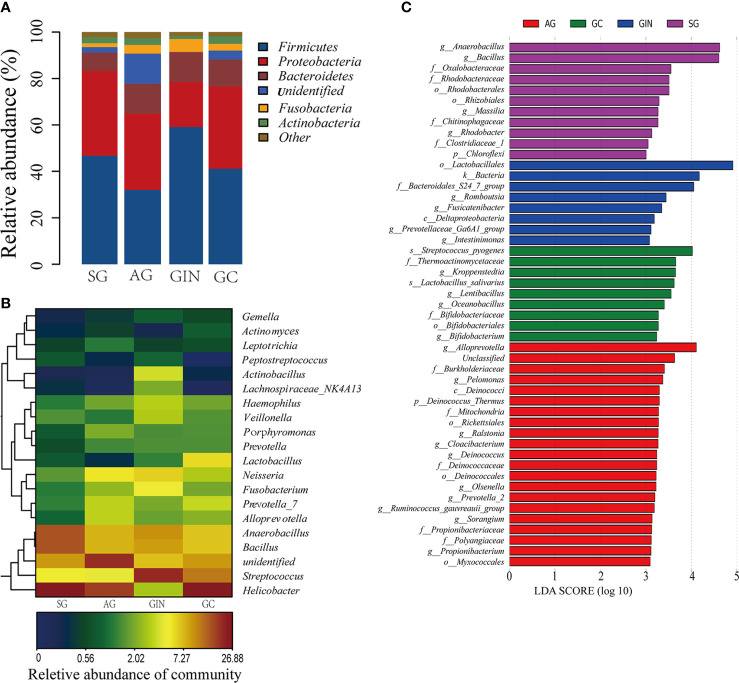
The microbiota composition in different groups. **(A)** Relative abundance of phyla in four groups. **(B)** Heatmap of 20 genera with the highest relative abundance in four groups. **(C)** Histogram of the linear discriminant analysis (LDA) scores for differentially abundant taxonomic features among four groups. Significance obtained by LDA effect size (LEfSe) at *p* < 0.05 (Kruksal–Wallis test) and LDA score>3 (f:phylum, c:class, o:order, f:family, g:genus, s:species).

The Relative abundance of phyla in four groups was shown in **Figure 2A**. The top 5 phyla in stomach were *Firmicutes*, *Proteobacteria*, *Bacteroidetes*, *Fusobacteria*, and *Actinobacteria*, with an average relative abundance of 44.75%, 31.04%, 11.32%, 3.49%, and 2.51%, respectively, in all the specimens. There was no significant difference among the four groups for each phylum. None of the phyla showed a continuous increasing or a decreasing changing trend from SG to GC.

There were a total of 561 identifiable genera in all specimens (**Supplementary Table 2**). Twenty genera with the highest relative abundance were shown in **Figure 2B**. *Helicobacter*, *Streptococcus*, *Bacillus*, and *Anaerobacillus* were the most abundant genera. With the progressive disease stages from SG to GC, none of these genera presented a continuous and consistent trend of change in abundance. The only four genera that continuously increased from SG to GC group were *Slackia*, *Selenomonas*, *Bergeyella*, and *Capnocytophaga*, although they had a relatively low abundance in stomach (**Table 1**).

**Table 1:** Relative abundance of selected genera in the four groups.

	Relative abundance (%)				*P^#^*
	SG	AG	GIN	GC	
**The genera enriched in GC compared with SG**					
*Prevotella_2*	0.014	0.317	0.299	0.135	0.006
*Oceanobacillus*	0.011	0.362	0.000	0.472	0.006
*Lentibacillus*	0.012	0.595	0.000	0.667	0.011
*Kroppenstedtia*	0.018	0.608	0.001	0.846	0.012
*Parvimonas*	0.076	0.177	0.500	0.178	0.020
*Erysipelotrichaceae_UCG-007*	0.194	0.248	0.258	0.242	0.021
*Staphylococcus*	0.079	0.115	0.013	0.113	0.026
*Eikenella*	0.024	0.015	0.022	0.232	0.028
*Bifidobacterium*	0.108	0.190	0.002	0.327	0.038
**The genera depleted in GC compared with SG**					
*Prevotella_1*	0.173	0.019	0.154	0.015	0.001
*CL500-29_marine_group*	0.086	0.003	0.045	0.008	0.001
*Rhodobacter*	0.285	0.011	0.063	0.014	0.003
*Massilia*	0.419	0.065	0.085	0.048	0.003
*Flavihumibacter*	0.153	0.001	0.055	0.013	0.003
*Ensifer*	0.108	0.028	0.059	0.019	0.003
*Hydrogenophaga*	0.073	0.004	0.026	0.005	0.003
*Anaerobacillus*	15.208	7.702	9.883	6.744	0.004
*Nitrospira*	0.176	0.014	0.047	0.017	0.004
*Ferruginibacter*	0.148	0.000	0.026	0.014	0.004
*Gemmobacter*	0.091	0.001	0.021	0.005	0.004
*Denitratisoma*	0.101	0.005	0.092	0.011	0.007
*Bacillus*	14.978	7.716	9.092	6.866	0.008
*Brevundimonas*	0.091	0.034	0.001	0.002	0.038
*Romboutsia*	0.156	0.011	0.609	0.136	0.043
**The genera enriched in GIN**					
*Romboutsia*	0.156	0.011	0.609	0.136	
*Fusicatenibacter*	0.089	0.002	0.492	0.002	
*Prevotellaceae_Ga6A1_group*	0.154	0.001	0.276	0.046	
*Intestinimonas*	0.027	0.014	0.270	0.035	
**The genera continuously enriched from SG to GC**					
*Slackia*	0.0000	0.0004	0.0007	0.0058	
*Selenomonas*	0.0280	0.0309	0.0393	0.0441	
*Bergeyella*	0.0135	0.0240	0.0269	0.0434	
*Capnocytophaga*	0.1540	0.2549	0.2727	0.4965	

^#^P: SG vs. GC.

LEfSe analysis was applied to identify the most relevant taxa responsible for the differences among disease stages (**Figure 2C**). We focused observations on the bacterial taxa with different abundance at genus and species level. The SG-enriched genera include *Anaerobacillus*, *Bacillus*, *Massilia*, and *Rhodobacter*. The AG-enriched genera include *Alloprevotella*, *Pelomonas*, *Ralstonia*, *Clocibacterium*, *Deinococcus*, etc. In GIN group, an enrichment in the genera *Romboutsia*, *Fusicatenibacter*, *Prevotellaceae-Ga6A1-group*, and *Intestinimonas* were observed. In GC group, enriched genera *Kroppenstedtia*, *Lentibacillus*, *Oceanobacillus*, and *Bifidobacterium*, and enriched species *Streptococcus pyogenes* and *Lactobacillus salivarius* were observed.

### Specific Bacterial Taxa Are Associated With Gastric Cancer and Gastric Intraepithelial Neoplasia

**Figure 3:**
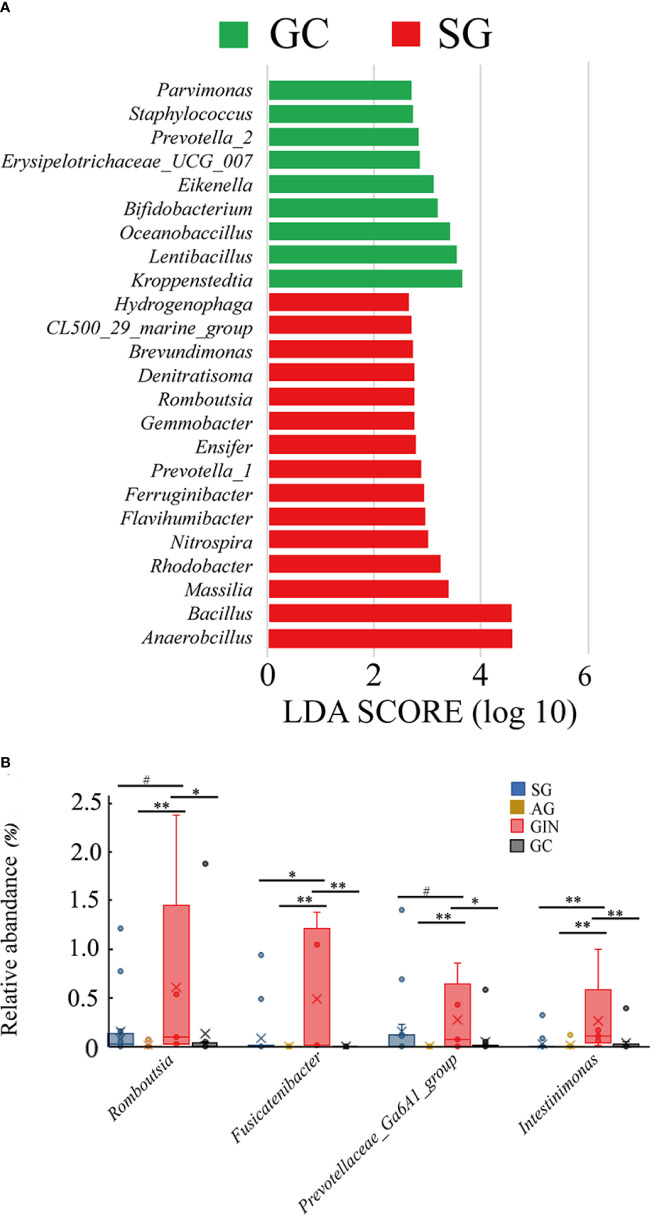
Specific genera associated with gastric cancer and gastric intraepithelial neoplasia. **(A)** Histogram of the linear discriminant analysis (LDA) scores for differentially abundant genera between gastric cancer (GC) and superficial gastritis (SG) groups. Significance obtained by LDA effect size (LEfSe) at *p* < 0.05 (Kruksal–Wallis test) and LDA score>2. **(B)** The relative abundance of GIN-enriched genera in four groups (Wilcoxon test, ^#^*p*≥0.05, *0.01≤*p*<0.05, **p<0.01).

When GC was compared with SG alone, LEfSe analysis (LDA>2) showed the potential taxonomic biomarkers on genus level in the two groups (**Figure 3A**). *Kroppenstedtia* (*p*=0.012), *Lentibacillus* (*p*=0.011), *Oceanobacillus* (*p*=0.006), *Prevotella-2* (*p*=0.006), etc. were more abundant in GC group, while *Anaerobacillus* (*p*=0.004), *Bacillus* (*p*=0.008), *Rhodobacter* (*p*=0.043), and *Massilia* (*p*=0.003), etc. were more abundant in SG group. We observed the changing trend of these genera along the disease stages and found that, none of them showed a continuous and consistent abundance change from SG to GC. However, *Prevotella_1*, *Massilia*, *Ensifer*, and *Bacillus* showed and a depleting trend from SG to GC with AG and GIN in between. *Kroppenstedtia*, *Lentibacillus*, *Bifidobacterium*, and *Oceanobacillus* showed an enriching trend in the groups of SG, AG, and GC (**Table 1**).

The gradual enriching pattern of some potential taxonomic biomarkers that were abundant in GC group seemed to be disturbed by GIN group. Were there specific changes in the microbiota community of GIN? LEfSe analysis revealed the taxa that differed GIN from other groups, and we focused on the genus level and noticed four GIN-enriched genera: *Romboutsia*, *Fusicatenibacter*, *Prevotellaceae-Ga6A1-group*, and *Intestinimonas*. Then we examined their changing pattern in the four disease stages (**Table 1**, **Figure 3B**). Wilcoxon test was used to compare the relative abundance of taxa between each two groups. All these four genera were significantly abundant in GIN group than in AG group, and *Fusicatenibacter* and *Intestinimonas* were significantly more abundance in GIN than in SG group. All of the four genera depleted significantly from GIN to GC group. This result suggested that the mucosal adjacent to GIN might provide a suitable growth environment for these bacteria, and their enrichment might be a marker of a precancerous condition.

### The Gastric Microbiota Profile Differs in Cardia Cancer and Non-Cardia Cancer

**Figure 4:**
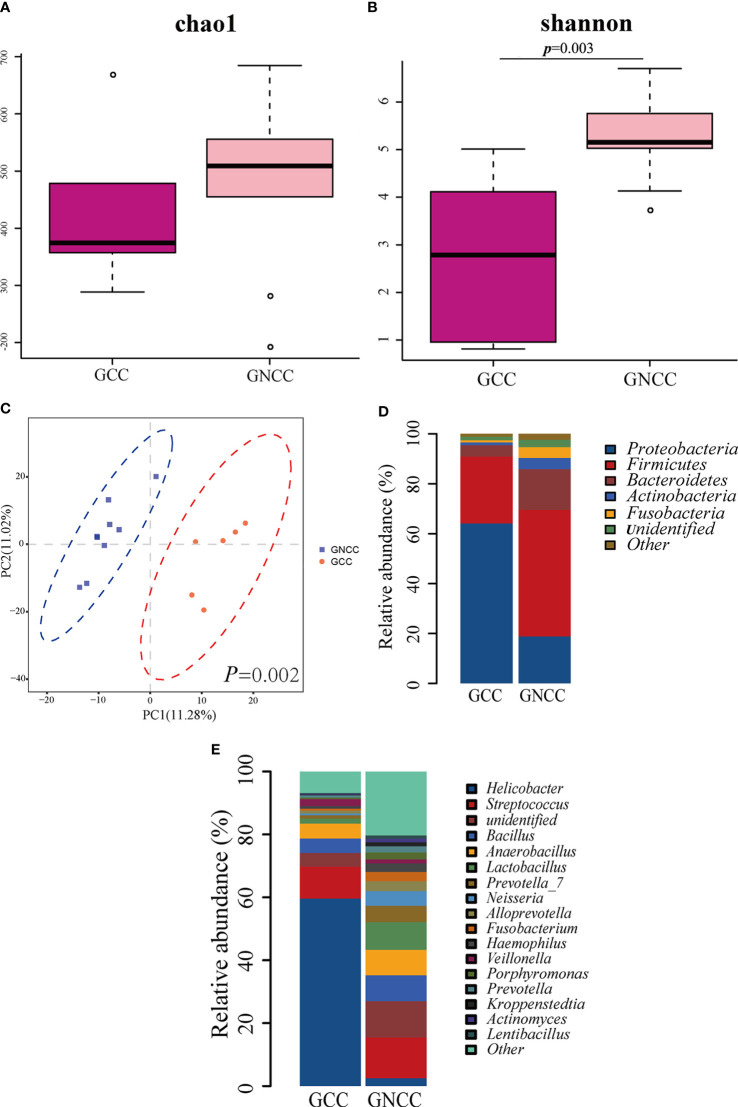
The different gastric microbiota profile between cardia cancer and non-cardia cancer. **(A)** There was no significant difference in Chao1 index between gastric cardia cancer (GCC) and gastric non-cardia cancer (GNCC) groups. **(B)** Shannon index was higher in GNCC group compared with GCC group (Student’s t-test was used to compare the two groups). **(C)** Partial least squares Discriminant Analysis (PLS-DA) revealed different microbial community structures between the two groups (ANOSIM was used to evaluate the similarity between groups). **(D)** Relative abundance of phyla in the two groups. **(E)** Relative abundance of genera in the two groups.

When we examined the GC group, we found that gastric cardia cancer (GCC), including patients GC1-6, and gastric non-cardia cancer (GNCC), including patients GC7-15, showed an obvious different microbiota profile. We thus divided GC group into GCC group and GNCC group, and compared their microbial structures. The patients’ age of GCC and GNCC were 66.5 ± 6.87 and 72.11 ± 13.38, respectively (*p*=0.39). We found that there was no significant difference in microbial richness between the two groups (Chao1 index, *p*=0.52, **Figure 4A**), however, microbial diversity was significantly higher in GNCC group (Shannon index, *p*=0.003, **Figure 4B**). PLS-DA at the OTU level revealed a significant separation of the two groups (ANOSIM, *p*=0.002), suggesting a striking different microbial community structures (**Figure 4C**). At phylum level, the abundance of *Proteobacteria* in GCC was significantly higher than in GNCC (0.641 *vs.* 0.189, *p*=0.018), while the abundance of *Bacteroidetes* and *Fusobacteria* were significantly lower in GCC than in GNCC (0.045 *vs.* 0.164, *p*=0.049, 0.008 *vs.* 0.043, *p*=0.036, respectively, **Figure 4D**). The comparison at genus level revealed that the high abundance of *Proteobacteria* in GCC was mainly due to the significant enrichment of *Helicobacter* in this group (**Figure 4E**). Species under the genus *Helicobacter* were not furtherly identified in this study.

## Discussion

The microbial structure in the stomach has been demonstrated changed during carcinogenesis. Most studies have found that gastric cancer is lower in both the microbial diversity and richness, compared with chronic gastritis (Francisco et al., 2014; Coker et al., 2018; Ferreira et al., 2018), while some other studies reported inconsistent results that the microbial diversity and richness increased in gastric cancer patients (Eun et al., 2014). In this study, no significant difference was found in the microbial diversity and richness between GC group and other groups, while the AG group showed the highest richness among the four groups. The different results of α diversity in different studies may be related to different age, gender and race of the patients. The results of β diversity in our study showed that SG, AG, and GC group could be separated well, which agreed with previous reports that there was a shift in the composition of microbial community along disease stages (Eun et al., 2014; Francisco et al., 2014; Coker et al., 2018).

We observed the microbial composition of the four disease stages. The five predominant phyla in all groups were *Firmicutes*, *Proteobacteria*, *Bacteroidetes*, *fusobacteria*, and *Actinobacteria*, and the major genera included *Helicobacter*, *Streptococcus*, *Bacillus*, *Anaerobacillus*, *Fusobacterium*, *Prevotella*, *Lactobacillus*, etc., which was consistent with the literature (Eun et al., 2014; Francisco et al., 2014; Emanuel et al., 2016; Coker et al., 2018).

To searching for potential taxonomic biomarkers that might related to carcinogenesis, we identified the most relevant taxa responsible for the differences among SG and GC by LEfSe analysis. At the level of genus, nine genera were abundant in GC, and 15 genera were depleted in GC. Among the GC-enriched genera, *Parvimonas*, *Eikenella*, and *Prevotella-2* were oral bacteria. *Parvimonas micra* in feces has been found related to colorectal cancer (Yu et al., 2017), and it is enriched in gastric mucosa in gastric cancer (Coker et al., 2018). The genus *Eikenella* contains only one species, *Eikenella corrodens*, which usually cause pneumonia, intra-abdominal abscess, or purulent skin and soft tissue infections from bites. This is the first study to find it related with gastric cancer. *Prevotella* has relatively high abundance in stomach, and several species in this genus have been found enriched in GC patients (Emanuel et al., 2016; Coker et al., 2018). *Kroppenstedtia*, *Lentibacillus*, and *Oceanobacillus*, which were from the environment including soil and water area, were also the first time to be found enriched in GC, and their roles in gastric carcinogenesis remain unclear. *Erysipelotrichaceae* has been reported to enrich in inflammatory bowel disease and colon cancer (Kaakoush, 2015). Its enrichment in GC implies its potential contribution to gastric carcinogenesis. To investigate the roles of these genera in carcinogenesis, we observed their changing trend in abundances in the four groups, but did not find a continuous increasing or decreasing trend in any of them. The only four bacterial taxa showing continuous increasing trend were *Slackia*, *Selenomonas*, *Bergeyella*, and *Capnocytophaga*. Interestingly, all of them were oral bacteria. The relationship between oral bacteria and gastric cancer has attracted much attention (Mirjana et al., 2020). Olabisi et al. observed significant centralities of OTUs corresponding to oral microbes *P. stomatis*, *Streptococcus anginosus*, *P. micra*, *S. exigua*, and *D. pneumosintes* in GC microbial ecology network and suggested them to be used as biomarkers to distinguish GC from SG (Coker et al., 2018). Sun et al. designed a scoring system for screening suspected gastric cancer patients by oral microbiome detection (Sun et al., 2018). Our results highlighted the significance of oral bacteria in gastric carcinogenesis, that these oral bacteria markers may serve as potential diagnostic tissue markers for GC.

Our study put GIN into disease stages to observe the microbial change in gastric carcinogenesis. According to the degree of cell atypia and structural disorder, GIN was divided into low-grade GIN (equivalent to mild to moderate dysplasia) and high-grade GIN (equivalent to severe dysplasia and carcinoma in situ) (Hamilton and Aaltonen, 2000). Srivastava et al. reported that low-grade GIN disappeared in 38%–75% of patients, remained unchanged for a long time in 19%–50% of patients, and developed into advanced gastric cancer in only a few patients (Srivastava and Lauwers, 2008). The specimens of GIN in our study were mainly taken from low-grade GIN patients (4/5), and the lesions were resected endoscopically due to the high risk of progression assessed by endoscopy (>2cm and/or demarcation line with irregular microsurface pattern) (Endoscopic Classification Review Group, 2005; Kim et al., 2014; Hwang et al., 2016). According to our observation, the β diversity analysis showed that GIN group did not separate well from other groups. When we observed the changing trend of the potential biomarkers, we found five out of nine GC-enriched genera presented with continuous enriching trend along SG to AG and to GC, but the trend was disturbed by GIN. The incompatibility of GIN group may due to the various prognosis of low-grade GIN.

The LEfSe analysis identified four GIN-enriched genera, and we investigated their roles in gastric precancerous disease. All of the four genera were intestinal bacteria that usually show protective effects. *Romboutsia* depletes significantly in colorectal cancer, and its absence is a sign of intestinal mucosal damage (Mangifesta et al., 2018). *Fusicatenibacter* belongs to *Clostridium. cluster XIVa* and contains only one species, *Fusicatenibacter saccharivorans* (Takada et al., 2013). Takeshita’s study revealed that the prevalence of *F.saccharivorans* was strikingly lower in active ulcerative colitis than quiescent ones, and suggested that human-derived *F.saccharivorans* can suppress intestinal inflammation, probably through IL-10 induction (Takeshita et al., 2016). *Prevotellaceae-Ga6A1-group*, which belongs to the *Prevotellaceae* family, is a short chain fatty acids (SCFAs)-producing bacterium. *Intestinimonas* is a butyrate-producing bacterium isolated from intestine (Klaring et al., 2013). SCFAs, including butyrate, play an important role in maintaining the intactness of intestinal epithelial cells and the normal function of the intestine. The significant enrichment of these genera in GIN compared with SG and AG may be due to a gastric niche favoring their colonization, and their depletion in GC may follow a similar path as observed in inflammatory bowel disease and colorectal cancer. It should be noted that the number of high-grade GIN in this study was very small, and the results of low-grade GIN may not represent the situation of high-grade GIN. The potential of these bacteria to be indicators of precancerous disease needs to be verified by further studies with more cases and validated by real-time quantitative PCR.

When the GC group was divided into GCC and GNCC, we found that the bacterial composition of the two subgroups were totally separated on PLS-DA. The significant higher abundance of *Helicobacter* in the GCC group corelated with the lower microbial diversity in this group and resulted in the difference in the bacterial structure between the two groups. The genus of *Helicobacter* was not further identified into species in our study. Han et al. investigated the presence of *Helicobacter* DNA in gastric cancer patients and revealed that the majority was *H. pylori* in human stomach. *H. cinaedi*, *H. mustelae*, and *C. hyointestinalis* also existed but rarely occurred (Han et al., 2010). So in our study we considered that the genus *Helicobacter* could be represented by *H. pylori*. *H. pylori* has been reported to alter gastric microbiome structure (Kienesberger et al., 2016). The abundance of *H. pylori* was found decreased from SG to GC in most studies (Eun et al., 2014; Ferreira et al., 2018; Hsieh et al., 2018; Liu et al., 2019). In this study, we found it depleted from SG to AG to GIN, but enriched in GC, as a result of high *H. pylori* abundance in the GCC group. The incidence rate of GCC is increasing in western countries (Devesa and Fraumeni, 1999; Brown and Devesa, 2002). However, the relationship between *H. pylori* infection and increasing risk of GCC is controversial (Egi et al., 2007; Hansen et al., 2007; Kamangar et al., 2006; Kim et al., 2012; Wang et al., 2014). A meta-analysis concluded that studies conducted in western countries tend to show a neutral or even negative association, while in eastern populations with high GC incidence including China, Korea, and Japan, there is evidence of a higher risk of GCC among the infected (Marlene et al., 2011). Gao et al. concluded from a study conducted in Southeast China that *H. pylori* may affect the gastric microbial structure in the pathogenesis of gastric cardia adenocarcinoma (Gao et al., 2019). But the mechanisms of non-*H. pylori* bacteria’s influence on GCC are still needs to be investigated. In this study, the abundance of *Helicobacter* was significantly higher in GCC than in GNCC, we infer that this may be attributed to the fact that the boundary of atrophic gastritis usually extends from gastric antrum to cardia, and the site suitable for *H. pylori* colonization also retreats from antrum to cardia. Further studies with more cases are needed to verify this point of view.

## Conclusions

Although our study is limited by the low number of patients with gastric intraepithelial neoplasia, we showed that the gastric microbial structure changed along SG-AG-GIN-GC stages. We found some bacterial taxa associated with GC and GIN, and their potential to be indicators of cancer and precancerous stages needs to be verified by further studies. We also found a significantly different microbial structure between the cardia cancer and non-cardia cancer groups characterized by a higher abundance of *Helicobacter* in the cardia cancer group, and suggested that gastric microbiota might play different roles in the carcinogenesis of cardia cancer and non-cardia cancer.

## Data Availability Statement

The original contributions presented in the study are publicly available. This data can be found here: https://www.ncbi.nlm.nih.gov/bioproject/PRJNA634837.

## Ethics Statement

The studies involving human participants were reviewed and approved by the Ethics Committee of Nanjing First Hospital. The patients/participants provided their written informed consent to participate in this study.

## Author Contributions

ZZ designed the study. XZ analyzed the data and wrote the initial manuscript. CL and WC performed sample collection and laboratory experiments. All authors contributed to the article and approved the submitted version.

## Funding

This work was supported by the Medical Science and technology development Foundation and the Nanjing Municipality Health Bureau (Grant number YKK16135 and YKK18103).

## Conflict of Interest

The authors declare that the research was conducted in the absence of any commercial or financial relationships that could be construed as a potential conflict of interest.

## Abbreviations

SG, Superficial gastritis; AG, Atrophic gastritis; IM, Intestinal metaplasia; GIN, Gastric intraepithelial neoplasia; GC, Gastric cancer; GCC, Gastric cardia cancer; GNCC, Gastric non-cardia cancer; *H. pylori, Helicobacter pylori;* ESD, Endoscopic submucosal dissection; OUT: Operational taxonomic unit; PLS-DA, Partial least squares discrimination analysis; ANOSIM, Analysis of similarity; LEfSe, Linear discriminant analysis effect size; LDA, Linear discriminant analysis.
