# Supplementary material for: Alterations of Gastric Microbiota in Gastric Cancer and Precancerous Stages
Source: Front Cell Infect Microbiol. 2021 Mar 3;11:559148. doi: 10.3389/fcimb.2021.559148 (PMC7966516; doi:10.3389/fcimb.2021.559148)
Supplement: Supplementary file 3 [file Table_3.docx]

Process of DNA extraction

Bacterial genomic DNA was extracted with the E.Z.N.A ®Stool DNA Kit (Omega Bio-tek, Norcross, GA, U.S.), following the manual.

1. Add up to 200 mg stool sample in a 2mL microcentrifuge tube and place the tube on ice.
2. Add 540 μl SLX-Mlus Buffer, vortex for 10 minutes or until the stool sample is completely homogenized.
3. Add 60 μl DS Buffer and 20 μl protein K solution, vortex or invert to mix thoroughly.
4. Incubate at 70 ℃ for 13 minutes. Vortex the sample twice during incubation.
5. Incubate at 95 ℃ for 5 minutes.
6. Add 200 μl SP2 Buffer, and vortex at maximum speed for 30 seconds.
7. Let sit on ice for 5 minutes, then centrifuge at a speed of 13000g for 5 minutes.
8. Transfer 400 μl cleared supernatant to a new 1.5ml microcentrifuge tube.
9. Add 200 μl cHTR Reagent, and vortex at maximum speed for 10 seconds.
10. Let sit at room temperature for 2 minutes, then centrifuge at a speed of 13000g for 2 minutes.
11. Transfer 250 μl cleared supernatant to a new 1.5ml microcentrifuge tube.
12. Add 250 μl BL Buffer and 250μl 100% ethanol, and vortex at maximum speed for 10 seconds.
13. Insert a Hibind® DNA Mini Column into a 2 ml Collection Tube.
14. Transfer all of the sample from STEP 12 to the Hibind® DNA Mini Column, then centrifuge at a speed of 13000g for 1 minute.
15. Discard the filtrate and transfer the Hibind® DNA Mini Column to a new 2 ml Collection Tube.
16. Add 500 μl VHB Buffer, centrifuge at a speed of 13000g for 30 seconds, discard the filtrate and reuse collection tube.
17. Add 700 μl DNA Wash Buffer, centrifuge at a speed of 13000g for 1 minute, discard the filtrate and reuse collection tube.
18. Repeat STEP 16-17, then centrifuge at a speed of 13000g for 2 minutes.
19. Transfer the column into a clean 1.5 ml microcentrifuge tube.
20. Add 100-200 μl Elution Buffer heated to 65℃ directly to the center of the HiBind® matrix, let sit at room temperature for 2 minutes.
21. Centrifuge at a speed of 13000g for 1 minute.
22. Store DNA at -20℃.
